# Mortality in Pharmacologically Treated Older Adults with Diabetes: The Cardiovascular Health Study, 1989–2001

**DOI:** 10.1371/journal.pmed.0030400

**Published:** 2006-10-17

**Authors:** Richard A Kronmal, Joshua I Barzilay, Nicholas L Smith, Bruce M Psaty, Lewis H Kuller, Gregory L Burke, Curt Furberg

**Affiliations:** 1 Collaborative Heath Studies Coordinating Center and Department of Biostatistics, University of Washington, Seattle, Washington, United States of America; 2 Kaiser Permanente of Georgia and Division of Endocrinology, Emory University School of Medicine, Atlanta, Georgia, United States of America; 3 Department of Epidemiology, University of Washington, Seattle, Washington, United States of America; 4 Departments of Medicine, Epidemiology, and Health Services, University of Washington, Seattle, Washington, United States of America; 5 Department of Epidemiology, University of Pittsburgh, Pittsburgh, Pennsylvania, United States of America; 6 Department of Public Health Sciences, Wake Forest University School of Medicine, Winston-Salem, North Carolina, United States of America; The George Institute, Australia

## Abstract

**Background:**

Diabetes mellitus (DM) confers an increased risk of mortality in young and middle-aged individuals and in women. It is uncertain, however, whether excess DM mortality continues beyond age 75 years, is related to type of hypoglycemic therapy, and whether women continue to be disproportionately affected by DM into older age.

**Methods and Findings:**

From the Cardiovascular Health Study, a prospective study of 5,888 adults, we examined 5,372 participants aged 65 y or above without DM (91.2%), 322 with DM treated with oral hypoglycemic agents (OHGAs) (5.5%), and 194 with DM treated with insulin (3.3%). Participants were followed (1989–2001) for total, cardiovascular disease (CVD), coronary heart disease (CHD), and non-CVD/noncancer mortality. Compared with non-DM participants, those treated with OHGAs or insulin had adjusted hazard ratios (HRs) for total mortality of 1.33 (95% confidence interval [CI], 1.10 to 1.62) and 2.04 (95% CI, 1.62 to 2.57); CVD mortality, 1.99 (95% CI, 1.54 to 2.57) and 2.16 (95% CI, 1.54 to 3.03); CHD mortality, 2.47 (95% CI, 1.89 to 3.24) and 2.75 (95% CI, 1.95 to 3.87); and infectious and renal mortality, 1.35 (95% CI, 0.70 to 2.59) and 6.55 (95% CI, 4.18 to 10.26), respectively. The interaction of age (65–74 y versus ≥75 y) with DM was not significant. Women treated with OHGAs had a similar HR for total mortality to men, but a higher HR when treated with insulin.

**Conclusions:**

DM mortality risk remains high among older adults in the current era of medical care. Mortality risk and type of mortality differ between OHGA and insulin treatment. Women treated with insulin therapy have an especially high mortality risk. Given the high absolute CVD mortality in older people, those with DM warrant aggressive CVD risk factor reduction.

## Abstract 2

The negative impact on mortality of diabetes persists into old age. Elderly people with diabetes might be twice as likely to die from CVD as people without diabetes. More aggressive treatment of CVD risk factors in older patients should be considered.

## Editors' Summary

**Background.**

Diabetes is a growing global health problem. By 2030, 300 million people worldwide may have this chronic, incurable disorder, double the current number. People with diabetes have dangerously high amounts of sugar in their blood. Blood-sugar levels are normally controlled by insulin, a hormone made by the pancreas that tells cells to absorb sugar from the blood. This control fails in people with diabetes, either because they make no insulin (type 1 diabetes) or because their cells are insensitive to insulin (type 2 diabetes). Type 1 diabetes is controlled with insulin injections; type 2 diabetes is controlled with diet, exercise, and pills that reduce blood-sugar levels. Long-term complications of diabetes include kidney failure, blindness, and nerve damage. Individuals with diabetes also have an increased risk of developing cardiovascular disease (CVD)—heart problems, strokes, and poor circulation—because of damage to their blood vessels.

**Why Was This Study Done?**

Epidemiological studies (investigations of disease patterns, causes, and control in populations) have indicated that diabetes increases the risk of death (mortality) from CVD in young and middle-aged people, but it is not known whether this is also true for old people. It is also not known what effect long-term treatment for diabetes has on mortality or whether the risk of death from CVD is decreasing in diabetic people as it is in the general US population. This information would help physicians provide health care and lifestyle advice to people with diabetes. In this study, the researchers have investigated mortality patterns in elderly diabetic people by looking at data collected between 1989 and 2001 by the US Cardiovascular Health Study, an observational study of nearly 6,000 people aged over 65 years (in this type of study participants are observed without imposing any specific changes to their lifestyle, behavior, medical care, or treatments).

**What Did the Researchers Do and Find?**

Participants were screened at the start of the Cardiovascular Health Study for CVD and diabetes (defined as drug-treated disease), for established CVD risk factors such as high blood pressure and smoking, for recently recognized CVD risk factors (for example, subclinical CVD), and for psychosocial factors associated with diabetes that might influence mortality, such as frailty and depression. At this time, about 5% of the participants were taking oral hypoglycemic agents for diabetes and about 3% were taking insulin. During the 11-year study, 40% of the participants died. After adjusting for CVD risk factors and psychosocial factors, the researchers calculated that people treated with oral hypoglycemic agents were 1.3 times as likely to die from all causes and people treated with insulin were twice as likely to die as people without diabetes. The risk of death from CVD was about twice as high in both groups of diabetic participants as in non-diabetic participants; the risk of death from coronary heart disease was increased about 2.5-fold. These adjusted relative risks are very similar to those found in previous studies. The researchers also report that participants treated with insulin were six times more likely to die from infectious diseases or renal failure than nondiabetic participants, and women treated with insulin had a particularly high mortality risk.

**What Do These Findings Mean?**

These findings indicate that the negative impact on mortality of diabetes persists into old age and that death from CVD is currently declining in both older diabetic people and nondiabetic people. In addition, they show that diabetic people treated with insulin are at a greater risk of dying relative to people without diabetes and those taking oral hypoglycemic agents. This might reflect the type of diabetes that these people had, but this was not investigated. How long participants had had diabetes was also not considered, nor how many people developed diabetes during the study. These and other limitations might mean that the reported excess mortality due to diabetes is an underestimate. Nevertheless, the estimate that elderly people with diabetes are twice as likely to die from CVD as people without diabetes is important. Many elderly people die anyway because of CVD, so this increased risk represents many more deaths than the similar increased risk in younger diabetic populations. Yet, elderly people often receive less-intensive management of CVD risk factors than younger people. The results of this study suggest that rectifying this situation could prolong the lives of many elderly people with diabetes.

**Additional Information.**

Please access these Web sites via the online version of this summary at http://dx.doi.org/10.1371/journal.pmed.0030400.

- MedlinePlus encyclopedia has pages on diabetes, heart disease, stroke and poor circulation
- The US National Institute of Diabetes and Digestive and Kidney Diseases provides patient information on diabetes
- Information for patients on prevention, diagnosis, and management of diabetes is available from the America Diabetes Association
- Patient information is available from the American Heart Association on all aspects of heart disease, including its association with diabetes
- Wikipedia pages on diabetes and cardiovascular disease (note that Wikipedia is a free online encyclopedia that anyone can edit)
- Further information is available about the Cardiovascular Health Study

## Introduction

Over the past several decades cardiovascular disease (CVD) mortality has decreased in the United States [1], while the prevalence of diabetes mellitus (DM) and its burden of CVD and coronary heart disease (CHD) have increased [2]. Whether people with DM have had a similar reduction in CVD mortality compared with individuals without diabetes is uncertain. A report from the Framingham Heart Study shows that absolute CVD mortality has decreased in people with DM, but that the mortality risk relative to individuals without DM remains unchanged (~2-fold), compared with the 1950s [3]. A report from New York City likewise shows that CHD mortality associated with DM has decreased in a similar measure to that of CHD without DM [4]. On the other hand, data from two other population studies suggest that CVD and CHD mortality in people with DM has actually increased, especially for women [5,6]. These four studies were done in predominantly middle-aged populations. The situation in older adults, in whom the incidence and prevalence of DM and CVD are highest [7], has been studied less fully. It is possible that the impact of DM is attenuated owing to the ubiquity of CVD and CHD in older age.

Population-based diabetes mortality studies have adjusted total and CVD mortality risk using traditional risk factors such as hypertension, age, sex, and smoking status (reviewed in [8]). Other factors associated with DM can influence mortality and serve as confounders. These include low levels of attained education [9,10], high rates of disability [11], depression [12,13], and frailty [14,15]. To date, no population-based study of DM mortality has adjusted estimates for these factors. CVD mortality is also influenced by several CVD risk factors that have been recognized and evaluated only in the last two decades. These include subclinical CVD [16] and elevated levels of inflammatory factors [17]. Few DM studies have adjusted mortality risk for these potential factors, and none simultaneously.

The Cardiovascular Health Study (CHS) is a longitudinal, observational study of adults aged 65 years or older. It has accrued more than 11 years of follow-up information in the current era of medical care with a high degree of detail. In the present study we describe mortality in older people with DM treated with oral hypoglycemic agents (OHGAs) and insulin. We adjust for traditional and nontraditional covariates to ensure that the severity of DM is not the explanation for differences that are found.

## Methods

Recruitment methods for CHS have been published [18]. In brief, a random sample of individuals, 65 y of age or above, derived from Medicare eligibility lists, and other household members 65 y or above, were invited to participate in the study. Potential participants were excluded if they were institutionalized, were unable to attend clinic visits, or had illnesses that were expected to lead to early death. Of those who were contacted, 9.6% were ineligible and 34.9% refused participation [18]. In total 5,201 participants were recruited in 1989–1990 and 687 in 1992–1993 to provide additional representation of African Americans. All participants signed informed consent forms upon entry into the study. Data for these analyses were from 1989 through 2001.

Upon entry into the study, participants were invited for a baseline interview. Information on prescription medications used in the preceding two weeks (including insulin and OHGAs) and psychosocial function were collected [19]. During a subsequent clinic visit, venipuncture was performed after an overnight fast. Plasma and serum were frozen at −70 °C, and shipped to the CHS Central Laboratory [20].

Following blood drawing, participants underwent the following cardiovascular tests: (1) resting ECG, (2) ankle and arm blood pressures, and (3) carotid artery ultrasounds [18]. Details of past medical history were obtained during the clinic visit.

### Definition of DM and Non-DM

For these analyses, DM is defined as pharmacologically treated disease (OHGAs and/or insulin) at baseline. Participants with fasting glucose (FG) over 125 mg/dl without a history of DM and not on hypoglycemic medications were not considered to have DM. There were 441 participants with no history of DM with baseline FG > 125 mg/dl. Of these participants, 155 (35.1%) received pharmacologic therapy during follow up. Preliminary analyses showed that the mortality of these participants differs from that of pharmacologically treated participants with DM at baseline and is the subject of another manuscript. In this paper these 441 participants were included in the “nondiabetic” group.

### Definition of Baseline CVD

Baseline clinical CHD was defined as a history of myocardial infarction, angina pectoris, or a revascularization procedure (coronary artery bypass grafting or percutaneous transluminal coronary angioplasty). CVD at baseline includes CHD and a history of stroke or transient ischemic attacks.

Isolated subclinical CVD was defined as the presence of any of the following in the absence of any clinical CVD: ankle/arm index below 0.9 in the absence of claudication or peripheral arterial disease surgery; major ECG changes in the absence of prevalent CHD; and common and internal carotid artery intima-media thickness in the upper 20% of the distribution or common carotid stenosis over 25%. These criteria have been used in prior CHS studies [16].

### Baseline Functional and Psychosocial Factors

Participants answered several standard interviewer-administered questionnaires on physical and cognitive function and psychosocial factors. Physical functioning was ascertained using a modified version of the Health Interview Supplement on Aging questionnaire [21], from which difficulties with activities of daily living (ADLs) and instrumental ADLs (IADLs) were derived. In addition, two questions regarding self-assessed quality of life were asked.

Depression was measured using the Center for Epidemiologic Studies Depression Scale [21]. A total of ten questions were asked about how the participant had felt in the preceding week. They were scored on a scale of 0 to 3. The higher the score, the more depressed was the person (range 0 to 30). Cognitive function was measured by the Mini-Mental State examination [22] and the Digit-Symbol Substitution test [23].

### Baseline Frailty

Baseline frailty was defined using a clinical phenotype that has been validated in the CHS [24]. This phenotype is met by having three or more of the following five measured criteria: (1) unintentional weight loss of more than 4.54 kg (10 lb) in the preceding year; (2) weakness defined as grip strength in the lowest 20% by gender and body mass index; (3) exhaustion by self-report of being tired most of the time or always with any effort, or stating “I cannot get going”; (4) slowness defined as the lowest 20% by gender and height for time to walk 4.6 m (15 ft); (5) low exercise tolerance as defined by the lowest 20% for males (<383 Kcal/wk) and females (<270 Kcal/wk), based on the short version of the Minnesota Leisure Time Activity Questionnaire [25]. Those with one or two criteria were considered “pre-frail,” an intermediate syndrome with increased risk for the development of frailty.

### Follow-Up and Ascertainment and Verification of Mortality

Participants were contacted every six months. Contacts alternated between a telephone interview and a clinic examination. At each contact, participants were asked about cardiovascular events and hospitalizations. Discharge summaries and diagnoses were obtained for all hospitalizations, including fatal events. Those who had died were often identified by family members or friends and reviews of obituaries. For potential incident cardiovascular events and death, additional information was collected, including cardiac enzymes, serial ECGs, death certificates, autopsy reports, interviews of next of kin, and physician questionnaires. All deaths were reviewed by the CHS Events Committee and classified into one of five major groups: (1) atherosclerotic CHD; (2) cerebrovascular disease; (3) atherosclerotic disease other than CHD (other ASCVD, such as abdominal aortic aneurysm or ischemic bowel); (4) other cardiovascular diseases (such as valvular heart disease or pulmonary embolism); and (5) all other deaths (with causes specified in text fields). The term “combined CVD mortality” is used to denote death from causes 1–4.

### Statistical Analysis

Kaplan-Meier curves were computed to estimate the survival curves for the various causes of death. Cox proportional hazards regression was used to estimate the hazard ratios (HRs) for diabetes status with and without adjustment for significant predictors of mortality determined by backward elimination from among the variables listed in Table 1. The log rank test was used to test the statistical significance of differences in survival as a function of diabetes status. Time-dependent interactions were included in the initial models to determine if the proportional hazards assumption of the Cox model were violated. In all instances, the resulting test of the interactions was nonsignificant, and the interaction terms were not included in the final models.

**Table 1:**
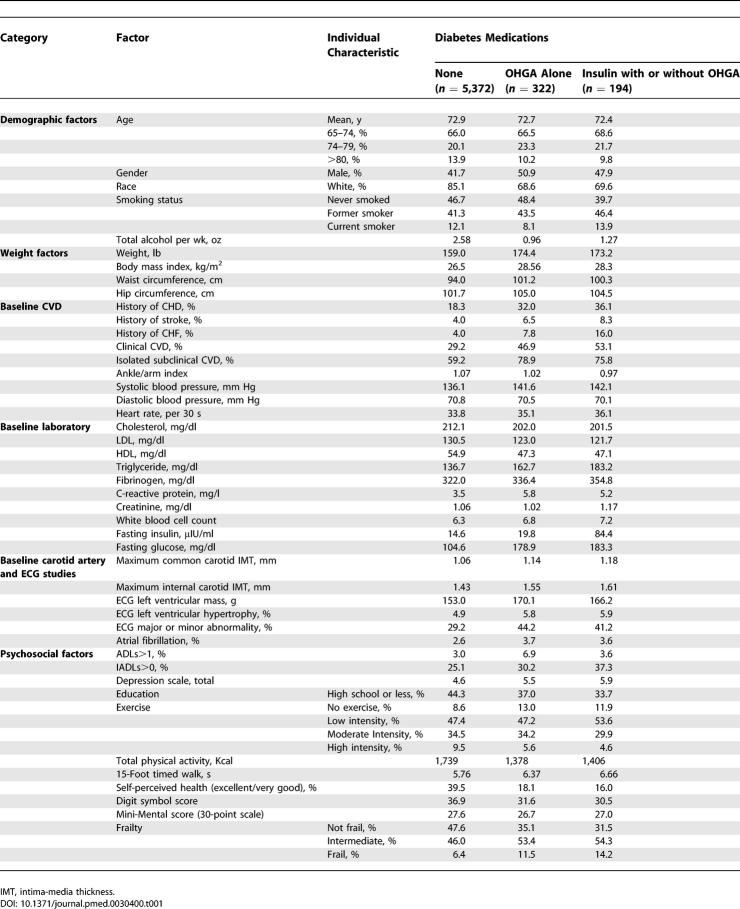
Baseline Factors of 5,888 Cardiovascular Health Study Participants Followed from 1989 to 2001 Categorized by Glycemic Status and Type of Antihyperglycemic Treatment

All analyses used Stata 8.0 (Stata, College Station, Texas, United States).

## Results

There were 5,372 participants without DM (91.2% of the cohort), 322 with DM treated with OHGAs (5.5%), and 194 with DM treated with insulin, with or without OHGAs (3.3%) (11 participants were on both insulin and OHGAs). Baseline characteristics are shown in Table 1. As compared with those with DM treated with either OHGAs or insulin, those without DM were more likely to be female; to be less centrally obese; to have less history of prior CHD, stroke, or CHF; and to have lower blood pressure and slower heart rate. They also had lower markers of inflammation, fewer difficulties with ADLs and IADLs, and were more likely to report good health. As compared with participants treated with OHGAs, those treated with insulin with or without OHGAs were more likely to smoke and to have a history of CVD and prevalent CVD.

During a median follow-up of 11.1 y (maximum 12.1 y) 2,391 (40.7%) of the 5,888 participants died. Causes of death are listed in Table 2. CHD mortality was higher among participants with DM. There were no large differences with regard to stroke death. Noncardiovascular causes of death, especially cancer, were more common among non-DM participants. Among OHGA-treated participants, deaths due to dementia and renal disease were common (~22% and ~8%, respectively), whereas among those treated with insulin therapy, infectious causes and renal disease were more common (~29% and ~13%, respectively).

**Table 2:**
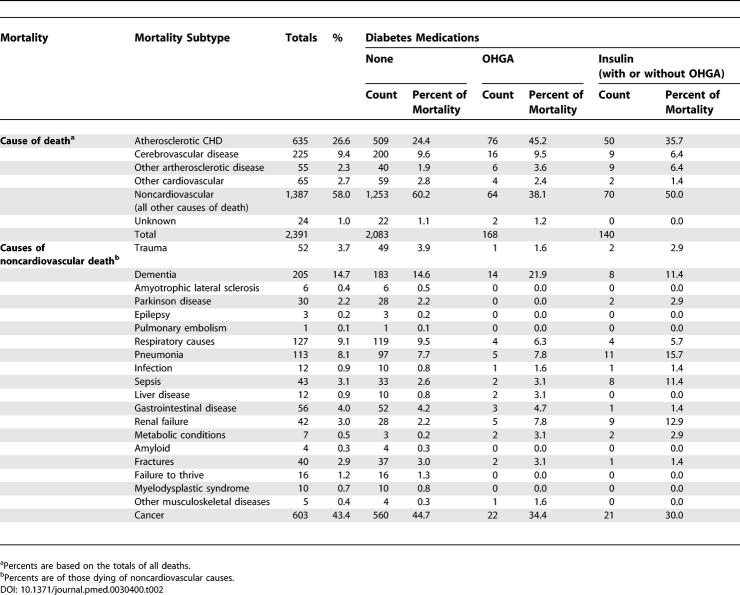
Causes of Death in CHS Participants Followed from 1989 to 2001 Categorized by Baseline Glycemic Status and Type of Antihyperglycemic Treatment

**Figure 1:**
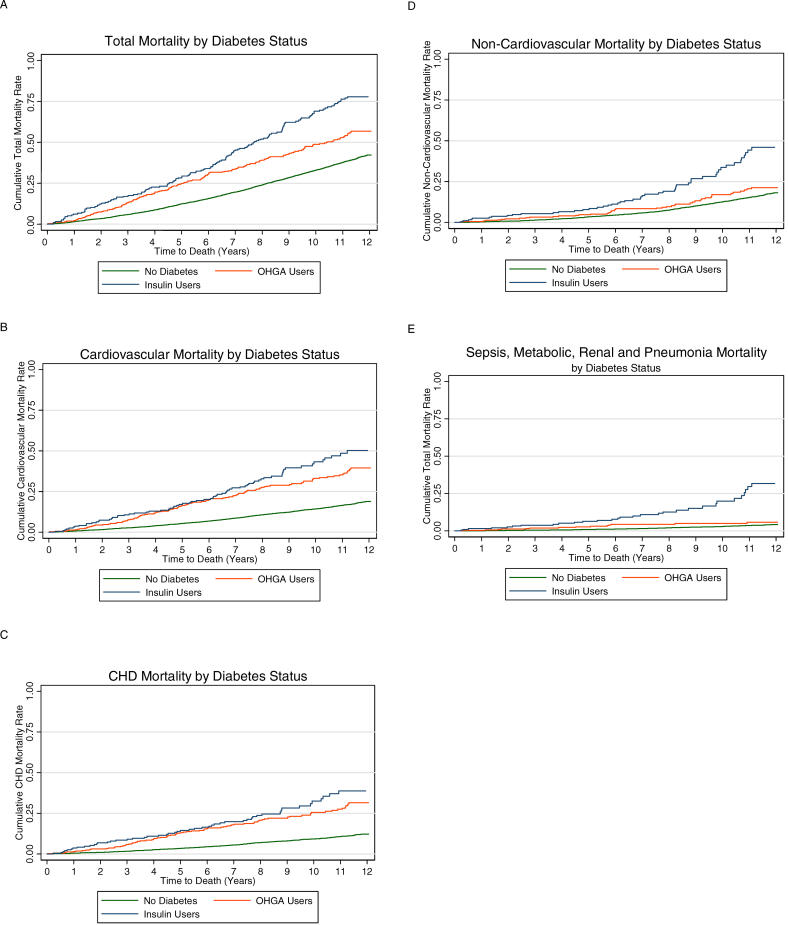
Mortality in Cardiovascular Health Study Participants According to Glycemic Status and the Type of Antihyperglycemic Treatment The cohort consisted of 5,372 participants without DM, 322 with DM treated with OHGAs, and 194 with DM treated with insulin with or without OHGAs followed from 1989 to 2001. (A) Total mortality of the cohort is plotted with regard to diabetes status. (B) Mortality due to CVD. (C) Mortality due to CHD. (D) Mortality due to noncardiovascular causes. (E) Mortality due to sepsis, metabolic derangement, and renal disease.

Cumulative mortality plots for total, combined CVD, and non-CVD endpoints are shown in Figure 1A. Median survival for those without DM was over 12 y. For those with DM treated with OHGA, median survival was slightly less than 10 y. For those treated with insulin, median survival was ~7.5 y, with 75% dead by 12 y follow-up. While most of the difference in mortality between those with and without DM was related to combined CVD (Figure 1B), especially CHD (Figure 1C), overall the non-CVD causes (Figure 1D)—especially infection, renal disease, and metabolic disease (Figure 1E)—accounted for a large percentage of the difference in survival.

Within the diabetes cohort, insulin-treated participants had higher total mortality than those treated with OHGAs. This difference was mostly accounted for by increased non-CVD mortality (especially renal, infectious, and metabolic causes; see Figures 1E and S1). There was little difference in combined CVD or CHD mortality in those treated with OHGAs or insulin. A test for nonproportional hazards (i.e., nonconstant HRs across time) was nonsignificant for the diabetes variable, suggesting that the apparent widening of the gap between the curves in Figure 1 may be due to chance.

Incidence rates for mortality endpoints, with unadjusted and adjusted HRs, are shown in Table 3 (a list of factors significantly associated with each endpoint can be found in Protocol S1). For most endpoints, incidence and relative risk increased stepwise between participants without DM and participants with DM treated with OHGAs and those treated with insulin. There were no differences for cancer. Unadjusted and age- and gender-adjusted relative risk estimates showed similar findings. Fully adjusted analyses, however, diminished the relative risk of death from stroke. Full adjustment attenuated the relative risk associated with diabetes category for total, combined CVD, and CHD mortality, and made the relative risk for non-CVD death significant only in DM participants treated with insulin.

**Table 3:**
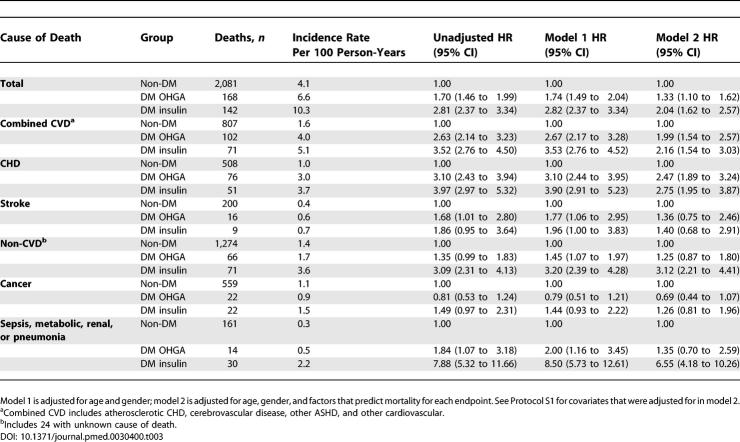
Incidence Rates and HRs for Total, CVD, and Non-CVD Endpoints in CHS Participants Categorized by Baseline Glycemia Status and Type of Antihyperglycemic Treatment

### Age and Gender Analyses

When a dichotomous variable for age (65–74 y versus >74 y) was entered into our models its interaction with diabetes status was not significant. There was, however, a highly significant interaction of gender with diabetes status (*p* < 0.001) (Table 4). Women with DM had a somewhat higher relative total mortality risk than women without DM (HR 2.28 [95% confidence interval (CI), 1.90 to 2.72]), as compared with the relative mortality risk of men with DM versus men without DM (HR 1.80 [95% CI, 1.63 to 2.11]). When analyses were limited to participants treated with OHGAs, HRs were almost identical (women, HR 1.62 [95% CI, 1.26 to 2.09]); men, HR 1.63 [95% CI, 1.33 to 2.00]). On the other hand, the relative mortality risk associated with insulin therapy was higher in women than in men. Women on insulin had the same mortality as men on insulin therapy (Figure S2).

**Table 4:**
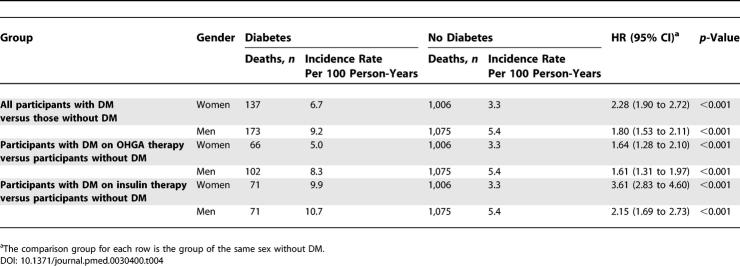
Incidence Rates and HRs for Total Mortality in Men and Women with Diabetes as Compared to Those without Diabetes from the Cardiovascular Health Study Followed 1989–2001

## Discussion

In this observational study from the era of current medical care we found that DM continues to be associated with a deleterious effect on mortality in older adults. The relative risk of total mortality for participants treated with OHGA and insulin relative to those without DM was 1.7 and 2.8, respectively. Adjustment for traditional CVD risk factors— age, gender, smoking, psychosocial factors (depression, frailty, and diminished cognitive function)—and newly recognized CVD risk factors (inflammation factors and isolated subclinical CVD)—decreased the relative risk to ~1.3 and 2.0, respectively. The total mortality risk estimate for OHGA users is lower than that of prior studies, while the estimate for insulin users is in line with prior studies [8]. These results emphasize the importance of assessing treatment type.

For combined CVD and CHD mortality, adjusted mortality risk was ~2 and 2.5 times higher (respectively) than in participants without DM. These estimates are similar to those from studies of older individuals with diabetes from prior decades, which adjusted for traditional CVD risk factors such as age, gender, smoking, and hypertension [8,26,27]. From our results two conclusions may be derived. First, given the decreasing rate of CVD and CHD mortality in the general population, but the unvarying relative risk of mortality associated with DM, it follows that older adults with DM are experiencing the same rate of decline in CVD and CHD mortality as people without DM. Second, the adjusted relative risks of CVD and CHD mortality that were similar to prior studies suggest that additional adjustment for subclinical CVD and inflammation as well as for psychosocial factors do not help explain the excess CVD and CHD mortality associated with DM. Only for stroke mortality, among measured CVD outcomes, was adjusted relative risk not significantly increased compared with people without DM and lower in incidence rate than that reported in prior decades. This result can be interpreted in several ways. It may signify that the many factors that we adjusted for accounted for differences in stroke risk. Alternatively, some of these factors are plausibly consequences of DM, and thus the decrease in risk associated with DM is a consequence of “overadjustment” for factors in the causal pathways leading to death. Also, our strict definition of DM (pharmacologically treated) most probably excluded people considered to have DM in prior studies. For example, only 6%–9% of the total diabetes mortality in this study was due to stroke, lower than the mortality attributed to stroke in prior studies [28,29]. Last, our stroke findings could be the result of type 2 error owing to the small number of stroke deaths.

With regard to age, participants with DM aged 75 years or older had similar relative mortality rates as those below 75 years of age, consistent with a study based on 1990s Medicare claims data, which demonstrated excess mortality risk from DM in every age group [30]. Given the high absolute CVD mortality in people over the age of 70 years, the relative risk of ~2–2.5 reported herein is far more noteworthy and of greater public health impact than a similar relative risk in a middle-aged population. Elderly people often receive less intensive management of CVD risk factors than younger individuals [31]. Our findings strengthen the rationale for the opposite approach.

As to gender, women with versus women without DM had a greater relative total mortality risk compared to men with versus men without DM (HR 2.28 [95% CI, 1.90 to 2.72] versus 1.80 [95% CI, 1.53 to 2.11]). When this risk was categorized by treatment type it was found that women treated with OHGAs had mortality risk similar to that of men, but a much higher relative mortality than men when treated with insulin therapy. Thus the overall increased mortality of women than men with DM appears to be accounted for by insulin therapy. This finding has not, to our knowledge, yet been reported. The increased relative mortality with insulin therapy for women with DM was due mainly to the lower risk of death in women without DM [26], since women and men with DM treated with insulin both had similarly high cumulative mortality (>75% at 12 y).

Limitations of this study should be noted. First, we may have overadjusted in our multivariable Cox models, since many of the factors examined may be in the causal pathway or strongly associated with causal factors that lead to the mortality, thus potentially underestimating the effect of diabetes. Nonetheless, our analyses demonstrate that differences in mortality exist between those with and without DM and between those taking and not taking insulin therapy, even after accounting for the presence or absence of such potent predictors of mortality as clinical and subclinical CVD, dementia, and functional status.

Second, our definition of DM required that pharmacological therapy be used. Those with FG over 125 mg/dl, but not on such therapy, were included in the group without diabetes. The result was to include in the nondiabetes cohort ~3% who went on to receive antidiabetes pharmacologic therapy and 8.2% who may have persisted in having elevated FG levels but were not on pharmacologic therapy. The effect of this would be to bias our estimates to the null and make them conservative. Our preliminary analyses indicate that mortality in these newly diagnosed DM participants is slightly less than that of participants with established DM. In addition, a substantial fraction of these patients with “new-onset” DM may not have had diabetes at all, in that when retested their glucose levels were within the nondiabetes range.

Third, CHS did not collect data on the duration of DM prior to enrollment. This is a deficiency, since duration of DM is an important factor in DM mortality. Participants on insulin, for example, may have had greater duration of DM. Nonetheless, factors that would be associated with diabetes duration—such as the extent of subclinical CVD and the presence of senescence and mobility disorders, e.g., frailty—did not explain the differences in mortality between those with or without DM and those treated and not treated with insulin. Finally, the entire CHS cohort was limited to people who were living at home, who were not institutionalized, and who were healthy enough (initially) to attend clinic visits. As such, our results are likely conservative estimates since DM most probably has a greater mortality effect in those with serious chronic illnesses.

In conclusion, DM continues to exert a strong negative impact on mortality in older adults in the current era of medical care. This effect is not explained by traditional and newer CVD risk factors. The negative effect of DM persists into advanced older age. Individuals, especially women, treated with insulin are at a much higher risk of death than those treated with OHGAs, especially from renal and infectious causes.

## Supporting Information

Figure S1 Mortality Curves Showing the Relative Proportions of Causes of Death by Glycemic Status and Type of Antihyperglycemic Treatment Mortality curves are based on the Cox models. The cohort consisted of 5,372 participants without DM, 322 with DM treated with OHGAs, and 194 treated with insulin with or without oral hypoglycemic agents followed from 1989 to 2001.
(237 KB DOC)

Figure S2 Cumulative Total Mortality Categorized by Gender, Diabetes Status, and Type of Antihyperglycemic Treatment Cohort included 5,372 participants without DM, 322 with DM treated with OHGAs, and 194 treated with insulin with or without oral hypoglycemic agents followed from 1989 to 2001.
(29 KB DOC)

Protocol S1 Statistically Significant Covariates on Cox Proportional Hazards Modeling for Table 3
(50 KB DOC)

## Abbreviations

ADL: activity of daily living
CHD: coronary heart disease
CHS: Cardiovascular Health Study
CVD: cardiovascular disease
DM: diabetes mellitus
FG: fasting glucose
HR: hazard ratio
OHGA: oral hypoglycemic agent
