# Supplementary material for: Mortality in Pharmacologically Treated Older Adults with Diabetes: The Cardiovascular Health Study, 1989–2001
Source: PLoS Med. 2006 Oct 17;3(10):e400. doi: 10.1371/journal.pmed.0030400 (PMC1609124; doi:10.1371/journal.pmed.0030400)
Supplement: Supplementary file 1 [file pmed.0030400.sd001.doc]

**PROTOCOL S1**

Statistically significant covariates on Cox proportional hazards modeling for **Table 3**.

- **I.	Total Mortality:** OHGA and insulin use vs no DM, increasing age, male gender, African American race, CHF at baseline, increasing maximum common and internal carotid intima media thickness, former or present smoker, increasing waist and hip/ waist ratio, increasing height, major ECG changes at baseline, increasing CRP level, poor self-rated health, decreasing digit symbol score, increasing pulse, difficulties with IADLs, increased LV mass, prevalent pre-frailty and frailty.
- **II.	Combined CVD Mortality:** OHGA and insulin use vs no DM, increasing age, male gender, African American race, increasing maximum common and internal carotid intima media thickness, LVH on ECG, increasing CRP level, decreasing digit symbol score, increasing pulse, increasing weight, major ECG changes at baseline, borderline and elevated blood pressure, difficulties with ADLs and IADLs, baseline pre-frailty and frailty, baseline CHF and CHD.
- **III.	CHD mortality:** OHGA use vs no DM, fasting insulin level < 150 uIU/ml in insulin users vs no DM, increasing age, male gender, African American race, baseline CHF, CHD, stroke, LVH on ECG, low energy expenditure, increasing LV mass, increasing weight, major ECG changes at baseline, decreasing digit symbol score, increasing maximum common and internal carotid intima media thickness, poor self-rated health, increasing CRP level.
- **IV.	Stroke Mortality:** OHGA and insulin use vs no DM, increasing age, male gender, African American race, increasing waist/hip ratio, increasing weight, increasing physical activity, increasing LDL cholesterol level, increasing maximum common carotid intima media thickness, decreasing digit symbol score, atrial fibrillation at baseline, prior stroke at baseline.
- **V.	Non**-**CVD Mortality:** OHGA use vs no DM, fasting insulin level < 150 uIU/ml in insulin users vs no DM, increasing age, male gender, former and current smoker, increasing waist, increasing hip/waist ratio and weight, decreasing education level, increasing HDL cholesterol level, baseline pre-frailty and frailty, increasing maximum internal carotid intima media thickness, decreasing digit symbol score, atrial fibrillation at baseline, and increasing depression score.
- **VI.	Sepsis, Metabolic, Renal, and Pneumonia Mortality:** OHGA use vs no DM, fasting insulin level < 150 uIU/ml in insulin users vs no DM, increasing age, male gender, CHD at baseline, increasing weight, difficulties with IADLs, increasing CRP level, increasing maximum internal carotid intima media thickness, increasing pulse, decreasing digit symbol score, decreasing diastolic blood pressure, increasing LV mass on ECG, major ECG changes at baseline, and prior stroke at baseline.
