# Supplementary material for: Mortality in Pharmacologically Treated Older Adults with Diabetes: The Cardiovascular Health Study, 1989–2001
Source: PLoS Med. 2006 Oct 17;3(10):e400. doi: 10.1371/journal.pmed.0030400 (PMC1609124; doi:10.1371/journal.pmed.0030400)
Supplement: Supplementary file 2 [file pmed.0030400.sg001.doc]

**Figure S1:** Mortality curves (based on the Cox models) showing the relative proportions of causes of death by glycemic status and type of antihyperglycemic treatment. The cohort consisted of 5372 participants without DM, 322 with DM treated with oral hypoglycemic agents, and 194 treated with insulin with or without oral hypoglycemic agents followed 1989-2001.

The figure illustrating competing causes of mortality was constructed by first fitting an unadjusted Cox model (as shown in Table 3) for each of the endpoints shown in the figure. From the Cox model, a smooth estimate of the survival curve is computed for each of the three groups (non-diabetic, OHGA user, insulin user) and for each of the three causes of mortality shown in the figure. The colored regions shown in each graph were computed based on the estimated mortality for the three causes of death shown in the figure. For example, the curve that defines the upper boundary for the red part of the figure for the non-diabetic participants would be equal to the 1- the survival curve for the sepsis, renal, metabolic and pneumonia curve times the survival curve for the CVD endpoint. The bottom of the red section in the same figure is 1-survival curve for the sepsis, renal, metabolic and pneumonia. The upper boundary for the blue portion of the curve is 1- product for the three survival curves corresponding to the three causes of death for the non-diabetics. The figure for each of the other categories (OHGA and insulin users) is computed in the same fashion. These figures allow one to see at a glance the relative importance of each cause of death for the three groups.
