# Supplementary material for: Mortality in Pharmacologically Treated Older Adults with Diabetes: The Cardiovascular Health Study, 1989–2001
Source: PLoS Med. 2006 Oct 17;3(10):e400. doi: 10.1371/journal.pmed.0030400 (PMC1609124; doi:10.1371/journal.pmed.0030400)
Supplement: Supplementary file 3 [file pmed.0030400.sg002.doc]

**Figure S2**: Cumulative total mortality categorized by gender, diabetes status, an type of antihyperglycemic treatment among 5372 participants without DM, 322 with DM treated with oral hypoglycemic agents, and 194 treated with insulin with or without oral hypoglycemic agents followed 1989-2001. OHGA = oral hypoglycemic agent
